# Repeated Observations Suggesting Kleptoparasitism by a Common Bottlenose Dolphin (*Tursiops truncatus*) on the Great Barrier Reef

**DOI:** 10.1002/ece3.74270

**Published:** 2026-09-13

**Authors:** Romney Edwards‐Francis, Jeremy J. Kiszka, Michael Ingram, Pieter Demmers, Jacinta Shackleton, Eric A. Ramos, Asia O. Armstrong

**Affiliations:** ^1^ School of Science, Technology, and Engineering University of the Sunshine Coast Sippy Downs Queensland Australia; ^2^ Department of Biological Sciences Institute of Environment, Florida International University Miami Florida USA; ^3^ Faculty of Science and Engineering Southern Cross University Lismore New South Wales Australia; ^4^ Independent Researcher Brisbane Queensland Australia; ^5^ Mote Marine Laboratory Sarasota Florida USA; ^6^ IUCN SSC Shark Specialist Group Dubai UAE

**Keywords:** behavioural plasticity, cetaceans, delphinids, foraging tactic, marine mammals

## Abstract

Kleptoparasitism, the deliberate theft of food from other animals, is a widespread but usually facultative foraging tactic used by many taxa. It has rarely been documented in aquatic species, particularly marine mammals. Here, we present observational evidence of kleptoparasitism initiated by a free‐ranging Common Bottlenose Dolphin ( *Tursiops truncatus* ) at Lady Elliot Island in the southern Great Barrier Reef, Australia. Between 2021 and 2025, opportunistic underwater observations and video recordings documented 11 kleptoparasitic interactions in which an individual dolphin targeted specific Bigeye Trevally ( *Caranx sexfasciatus* ), pursued them until regurgitation occurred, and consumed the expelled prey or prey remains while the trevally swam away. Using photo‐identification, all observations were confirmed to involve the same individual dolphin. The dolphin produced intense echolocation click trains and low‐frequency tonal sounds (LFTS, mean duration = 0.88 ± 0.51 s, range = 0.36–2.14 s). Based on video samples analysed, following pursuit and the production of clicks and LFTS, the trevally systematically regurgitated its prey, which was subsequently consumed by the dolphin. These observations represent the first detailed description of kleptoparasitism in cetaceans, and we speculate that induced regurgitation may function as an energetically efficient alternative to independent predation. The findings highlight the behavioural plasticity of delphinids and underscore the likelihood that rare or novel foraging tactics in cetaceans remain underreported due to observational limitations.

## Graphical abstract

This study documents the first detailed case of kleptoparasitism in cetaceans, with observations of a Common Bottlenose Dolphin repeatedly pursuing Bigeye Trevally to induce the fish to regurgitate their prey, which is then consumed by the dolphin. Photo‐identification confirmed all interactions involved the same individual and we speculate this foraging tactic may provide an energetically efficient alternative to direct predation. The study highlights the behavioural plasticity of dolphin foraging tactics and the likelihood that such novel strategies may remain underreported.

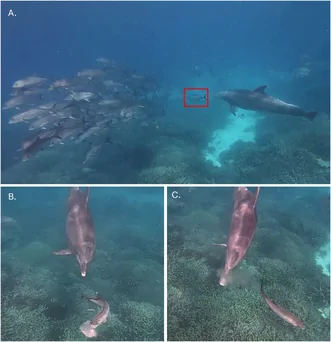

## 1 Introduction

Animals have evolved a range of foraging strategies and tactics that optimise energy intake while minimising foraging costs. According to optimal foraging theory, animals maximise their energy return by making decisions about what to eat, where to forage and what foraging tactics to use (MacArthur and Pianka 1966; Pyke et al. 1977; Stephens et al. 2007). While many species acquire resources by directly consuming prey or plants, others have evolved exploitative strategies that capitalise on foraging efforts produced by other animals. The best example of this is kleptoparasitism, which is found in a broad range of taxonomic groups, including birds, fish, mammals, reptiles and various groups of invertebrates (Iyengar 2008).

Kleptoparasitism is described as a form of exploitative competition that involves stealing food items already procured by another individual (Brockmann and Barnard 1979). It typically benefits the thief to the detriment of the host, as it incurs an energetic cost to the host from both the loss of food and the efforts taken to procure it (Iyengar 2008). While widespread, the development of kleptoparasitism is more likely under certain ecological and behavioural conditions. For example, the hosts typically congregate in large concentrations with regularity, so that there is a predictable food source for the kleptoparasite to exploit, and the kleptoparasite is an opportunistic feeder who has the ability to detect and obtain the food with great enough success to make it a profitable tactic (Brockmann and Barnard 1979; Iyengar 2008). Learning has also been identified as an important trait for assessing the trade‐offs associated with stealing food rather than foraging independently (Iyengar 2008; Morand‐Ferron et al. 2007). The fitness benefits derived from kleptoparasitism are not fully understood, but it is assumed that the net gain in stealing food is greater than the costs of independent foraging (Iyengar 2008; Triplet et al. 1999). As such, it is facultative in most species (Flower et al. 2012) and may be more prevalent when other food sources are scarce (Brockmann and Barnard 1979). Kleptoparasitism has most commonly been documented in birds, particularly seabirds (Brockmann and Barnard 1979; Flower et al. 2012; Iyengar 2008). In Roseate Terns (*Sterna dougalii*) (Shealer and Spendelow 2002), Common Terns ( *Sterna Hirundo* ) (García et al. 2011), and Magnificent Frigatebirds ( *Fregata magnificens* ) (Austin et al. 2019) kleptoparasitism occurs close to nesting sites during breeding, as an alternative to offshore foraging, and could result in reduced foraging costs through the exploitation of a predictable food source that has lower movement requirements (Austin et al. 2019). In these studies, this behaviour was only observed in some individuals, demonstrating that kleptoparasitism is facultative and not necessarily used across an entire species or population (García et al. 2011; Shealer and Spendelow 2002). Birds are likely ideally suited to this tactic, as the ability to move in three‐dimensional space allows manoeuvring around a target with speed and agility that is likely favourable for thieving behaviour (Iyengar 2008).

Underwater environments similarly allow manoeuvrability in three‐dimensional space, although kleptoparasitism is rarely reported in marine vertebrates. It could also be more prevalent in food‐limited environments, particularly in species with high metabolic rates such as marine mammals, which are dependent on predictable resources (Kiszka et al. 2015). The species it has been observed in are largely aquatic predators, such as reef sharks (*Carcharhinus* spp.) (Labourgade et al. 2020), European Eels ( *Anguilla anguilla* ) (Geffroy et al. 2016) and cichlid fish (Dominey and Snyder 1988). The rarity of such reports may be due to limitations of observing such interactions in underwater environments, rather than it representing a truly rare foraging tactic for aquatic species.

In cetaceans, there are only two reports in the literature documenting possible kleptoparasitic interactions among species, both involving the targeting of Sperm Whales ( *Physeter macrocephalus* ) by False Killer Whales ( *Pseudorca crassidens* ), Risso's Dolphins ( *Grampus griseus* ), and Northern Right Whale Dolphins ( *Lissodelphis borealis* ) (Palacios and Mate 1996; Smultea et al. 2014). Kleptoparasitism was suspected in both reports, based on the observation that Sperm Whales were seen regurgitating in both cases. However, no subsequent ingestion of regurgitated prey by the aggressors was observed, so the occurrence of kleptoparasitism in cetaceans remains unverified. Regardless, cetaceans are known for their diverse and adaptive foraging tactics, and their capacity for learning could facilitate the development of kleptoparasitism under favourable ecological conditions (Iyengar 2008; Rendell and Whitehead 2001). Bottlenose dolphins (*Tursiops* sp.) likely exhibit the most diverse range of foraging tactics observed in any small cetacean species with examples including mud ring feeding (Engleby and Powell 2019; Pierry et al. 2024), crater feeding (Kaplan et al. 2019), cooperative fish herding (Gazda et al. 2005), barrier feeding (Weiss 2006) and opportunistic depredation of gill nets (Read et al. 2003). Some populations also use both fixed human infrastructure and ships (Pace and Pedrazzi 2025; Weinpress‐Galipeau et al. 2021), or engage in cooperative foraging with fishermen (Bezamat et al. 2021). The diversity of foraging tactics employed by bottlenose dolphins demonstrates a behavioural plasticity and physical agility that could allow the emergence of kleptoparasitism within individuals of the species, particularly if it provides a less costly alternative to independent foraging (Sargeant et al. 2007).

Here, we present evidence that kleptoparasitism represents a foraging tactic for a Common Bottlenose Dolphin ( *Tursiops truncatus* ) in the southern Great Barrier Reef, Australia. We combined underwater behavioural observations with acoustics to document the first direct evidence showing that Common Bottlenose Dolphins may engage in kleptoparasitic interactions with other marine predators.

## 2 Methods

Opportunistic observations were made off Lady Elliot Island (−24.1149° S, 152.7163° E), a remote 0.35 km^2^ coral cay surrounded by an 84‐ha reef flat, located ~80 km off the Australian mainland. Observations were collated by the authors from opportunistic encounters while snorkelling. Observations were predominantly made by the authors, with several additional videos collected from resort guests, and were recorded using underwater action cameras (i.e., GoPro 9 or 10). All observations took place off the reef flat on the western side of the island, in water depths < 10 m, and within 100 m of the shore.

### 2.1 Photo‐Identification

Photo‐identification was used to identify the Common Bottlenose Dolphin observed in this study. Photo‐identification of small cetaceans is based on the permanent nicks and notches present on the dorsal fin, particularly the trailing edge (Würsig and Jefferson 1990). The same individual can be identified across years based on fin shape and scars that remain relatively unchanged over time.

### 2.2 Underwater Observations and Video Recordings

Video footage of foraging records was compiled and analysed by viewing clips in VLC Media Player and taking screenshots of key behaviours (VideoLan, Version 3.0.20). Feeding events were identified in the video recordings when the dolphin exhibited targeted chasing of an individual trevally that resulted in the trevally regurgitating food, followed by the dolphin eating the regurgitate (Table 1). The video footage was used for the development of an ethogram to describe behavioural states, define behaviours within these states, and to identify events in which the dolphin ingests regurgitated food in a manner that is consistent with kleptoparasitism (Table 2).

**TABLE 1:** Foraging records and feeding events documented between 2021 and 2025 by an adult male Common Bottlenose Dolphin ( *Tursiops truncatus* ).

Year	Date	No. of foraging records	No. of feeding events	Video duration (mm:ss)
2021	28 August	4	4	33:11
2022	6 June	3	1	05:51
2024	26 October	3	2	06:34
2025	10 October	7	3	02:45
2025	16 October	3	1	02:36

*Note:* Feeding events were recorded when the dolphin's target (Bigeye Trevally, *Caranx sexfasciatus* ) was observed regurgitating, and the dolphin ate the regurgitate.

**TABLE 2:** An ethogram of foraging behaviours that result in kleptoparasitism observed from underwater video footage of an adult male Common Bottlenose Dolphin ( *Tursiops truncatus* ) at Lady Elliot Island, Queensland, Australia.

State	Behaviour	Observation
Swimming	Travel	Regular directional movement at a constant speed
Foraging	Scanning	Slow movement with sharp turns. The head moves from side to side and echolocating clicks can be heard
	Targeting	Movement of the head becomes focused on a single individual fish and no longer moves from side to side
	Chasing	The focal individual accelerates, pursuing a single individual fish at greater speeds than during scanning or targeting. The individual maintains proximity to the target and mirrors the fish's movements with consistent directional changes
	Rushing	The focal individual lunges quickly at one or more fish, but regurgitation does not occur
Ingestion	Grasping prey	The targeted fish regurgitates, and the focal individual grasps the regurgitated food in its mouth
	Eating prey	The focal individual ceases chasing the fish and consumes the regurgitated food

### 2.3 Acoustic Data

Dolphin vocalisations were extracted from high‐quality video footage collected from a GoPro 9 video (28 August 2021) and were exported as a .wav file to characterise general patterns and parameters of vocalisations. Spectrograms were generated (Hamming window; 1024 samples; 90% overlap; 3 dB filter bandwidth: 47.7 Hz) and reviewed in Raven 1.6, and selection boxes were drawn around all dolphin vocalisations. The following frequency and time measurements were included: begin time (s), end time (s), duration (ms), inter‐signal interval (ms), minimum frequency (Hz), maximum frequency (Hz), delta frequency (Hz) and peak frequency (Hz). The number of tonal calls in a bout and the bout length were described. Calls were categorised as (1) echolocation: presence of click trains, or (2) low‐frequency tonal sounds (LFTS). LFTS are narrow‐band tonal vocalisations at lower frequencies than whistles and can be treated as a distinct subtype within the broader social‐acoustic repertoire rather than as echolocation or typical burst‐pulse sounds (Falkner et al. 2023).

## 3 Results

**FIGURE 1:**
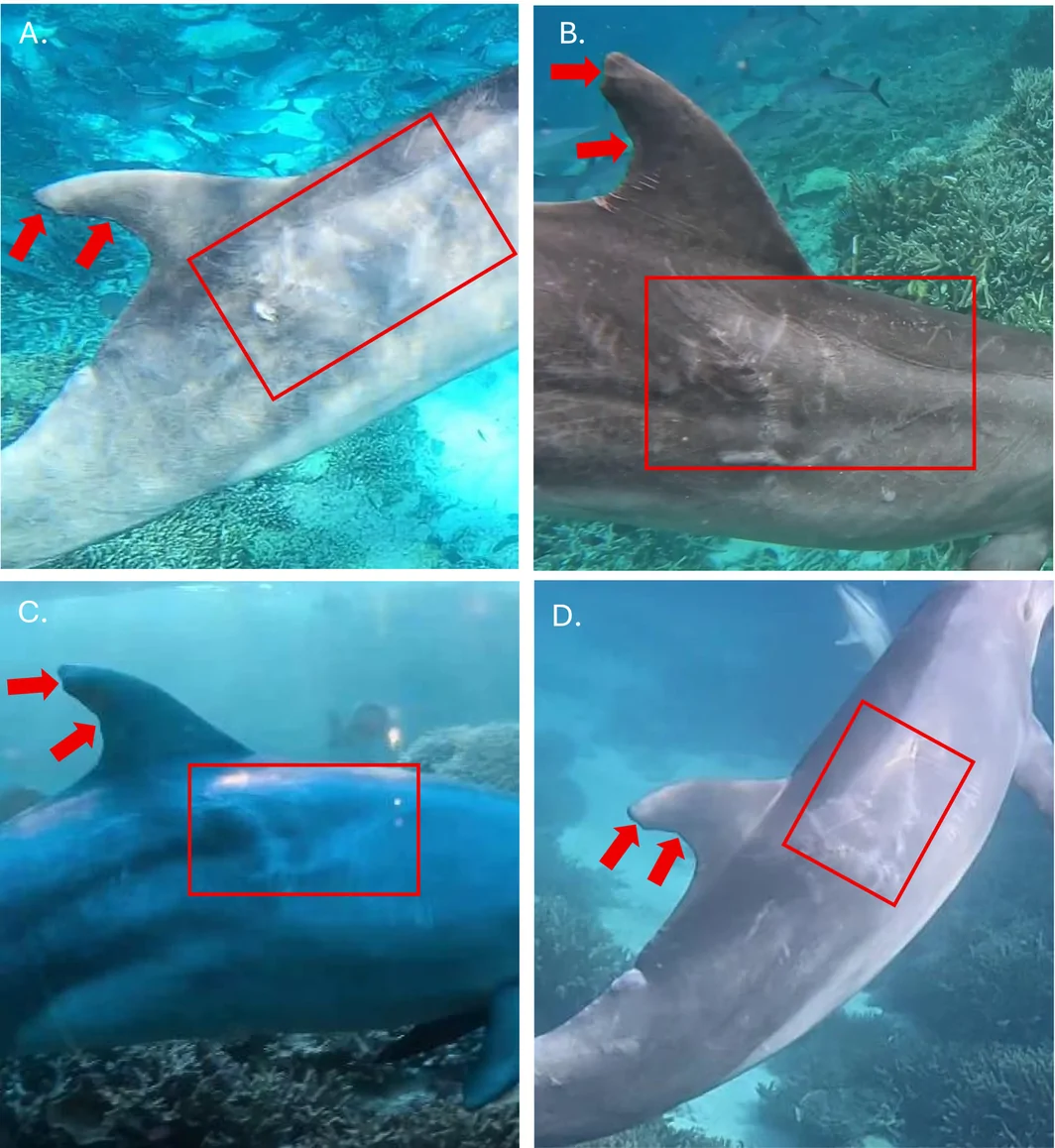
Photographs of ‘Bubbles’, a Common Bottlenose Dolphin ( *Tursiops truncatus* ) identified using photo‐identification. The individual can be identified by the distinctive scarring pattern on its right flank, indicated by a red box, and the unique notches in the dorsal fin, indicated by arrows. (A) 6 July 2022 (photo credit Jayne Jenkins); (B) 26 October 2024 (photo credit Pieter Demmers); (C) 10 October 2025 (photo credit Victoria Kronsell); and (D) 16 October 2025 (photo credit Michael Ingram).

Between June 2021 and October 2025, there were 20 documented foraging records of an adult male Common Bottlenose Dolphin interacting with schools of Bigeye Trevally ( *Caranx sexfasciatus* ) (Table 1). Eleven of the records resulted in feeding events, during which the dolphin ate food regurgitated by a trevally. Using underwater photo‐identification, a single individual was identified in each recording. Notches in this individual's dorsal fin and the distinctive V‐shaped scar on its right flank identified the dolphin in each observation as the same individual (Figure 1). This individual is regularly observed by Lady Elliot Island Eco Resort staff (informally known as ‘*Bubbles*’) and has been seen frequenting the western side of the island since at least 2017. Anecdotally, the individual has been known to pursue Bigeye Trevally in this area with regularity, and it is estimated that this individual is observed exhibiting this behaviour for 4–5 consecutive days at least twice a month. The dolphin was identified as male based on the distance between the genital slit and anus, and lack of mammary slits (Smolker et al. 1992).

**FIGURE 2:**
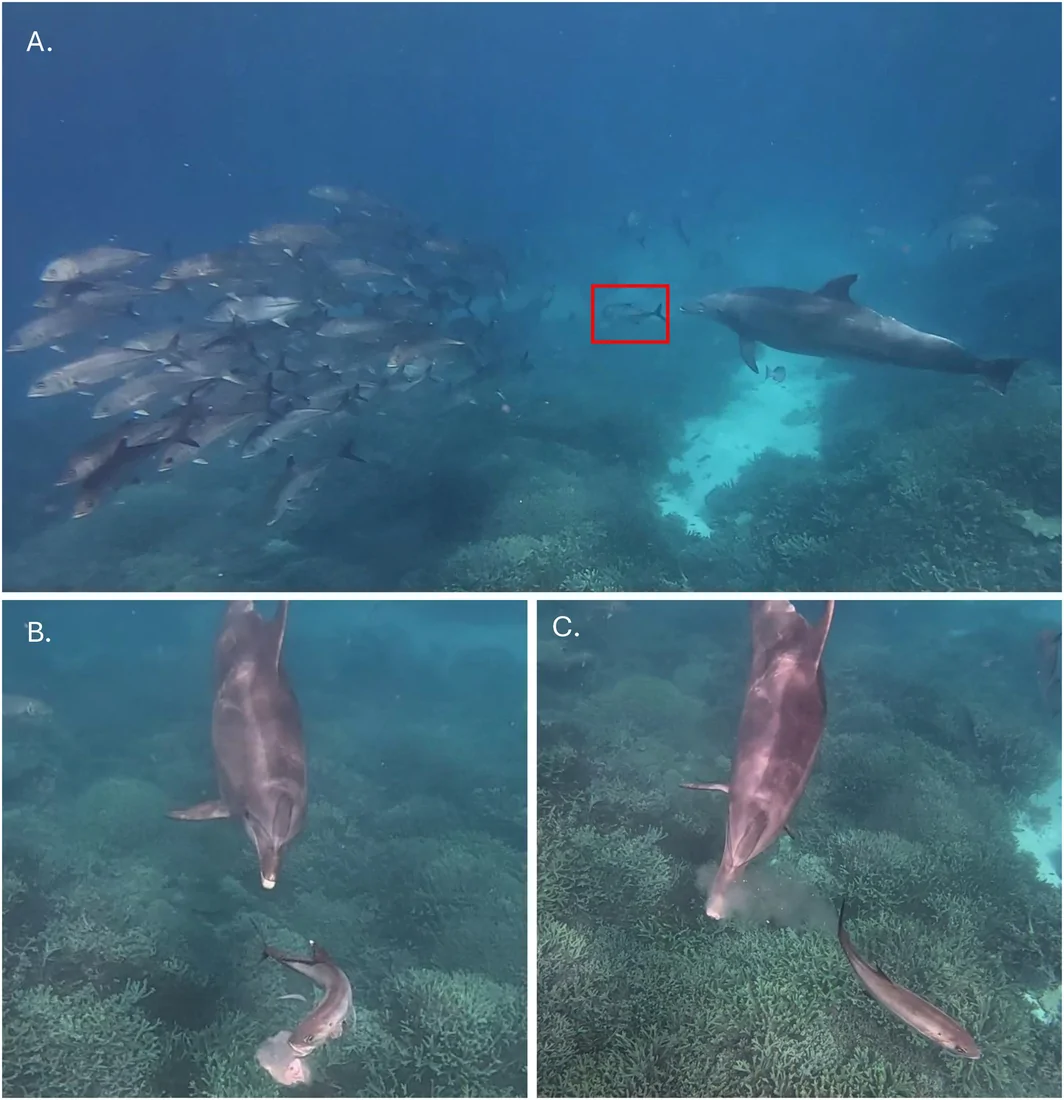
Documentation of an adult male Common Bottlenose Dolphin ( *Tursiops truncatus* ) eating the regurgitate of a Bigeye Trevally ( *Caranx sexfasciatus* ). (A) The dolphin targets a specific individual from the school of trevally, indicated by a red box. (B) The dolphin chases the trevally until it regurgitates. (C) The dolphin eats the regurgitated food while the trevally swims away (video credit Michael Ingram, Video 1).

Based on the 20 sightings, three behavioural activity states and seven individual behaviours were described in an ethogram (Table 2). Interactions between the dolphin and trevally began with Scanning, in which the dolphin was seen to move its head from side to side while echolocating, potentially suggesting active identification of a suitable target. Scanning was followed by Targeting, in which the dolphin appeared to focus on a specific individual trevally by isolating and pursuing the same individual, while appearing to ignore the rest of the school (Figure 2). The dolphin's head was directed towards the individual, and its movements usually increased in speed with frequent change of direction, mirroring the directional movement of the fish. This behaviour was followed by Chasing, in which the dolphin followed the trevally in close proximity. This behaviour ended when the trevally regurgitated, allowing the trevally to swim away while the dolphin ate the regurgitate (Figure 2, Video 1). This final stage falls under Ingestion in the ethogram: periods when the dolphin was observed grasping or consuming regurgitated food (Table 2).

**VIDEO 1:** Example of a kleptoparasitic foraging sequence by a Common Bottlenose Dolphin (*Tursiops truncatus*) to a school of Bigeye Trevally (*Caranx sexfasciatus*) (video credit Michael Ingram). Video content can be viewed at https://onlinelibrary.wiley.com/doi/10.1002/ece3.74270.

Following ingestion, the dolphin would return to the school of trevally, and the process would be repeated multiple times (observed up to 10 times over 30 min). During foraging events that resulted in ingestion, the dolphin appeared to target a fish and pursued that fish until it regurgitated, seemingly ignoring other trevally in proximity to the target. When the dolphin surfaced to breathe, visual tracking of the dolphin's focus revealed that it appeared to resume targeting the same fish as prior to surfacing. This behaviour was only observed being performed on Bigeye Trevally; no other species were observed being targeted in these interactions. In addition, the dolphin was not observed feeding on the Bigeye Trevally themselves, or any other prey. Although the dolphin has been seen with conspecifics, the kleptoparasitic interactions were always observed as a solitary behaviour, not a group foraging tactic. However, the individual was identified from video footage in the company of another adult and calf on one occasion on 12 July 2018, interacting with Bigeye Trevally and potentially exhibiting Scanning behaviour that is indicative of foraging. No feeding was observed during this encounter, so it is unclear whether this observation was an example of group foraging resulting in kleptoparasitism. Without evidence of other Common Bottlenose Dolphins exhibiting kleptoparasitic behaviours, this would seem to be a specialisation developed by a single individual.

**FIGURE 3:**
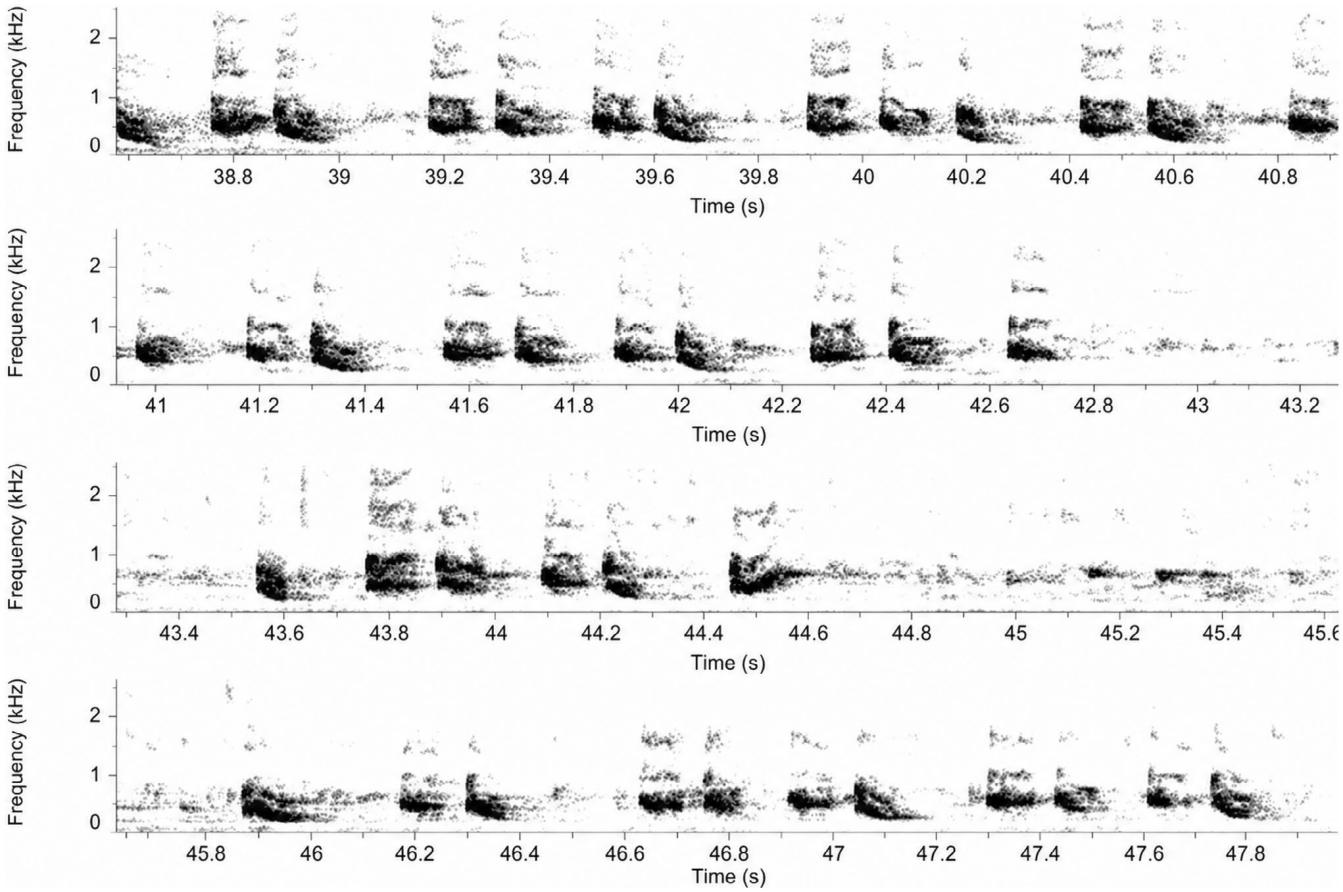
Spectrograms of Common Bottlenose Dolphin ( *Tursiops truncatus* ) low frequency tonal sounds recorded at Lady Elliot Island, Queensland, Australia. Spectrograms were created in Raven 1.6 with the following parameters: Hamming window; 1024 samples; 90% overlap; 3 dB filter bandwidth: 47.7 Hz.

In the video footage that captured high‐quality vocalisations for analysis, the male Common Bottlenose Dolphin produced 200 vocalisations for a total duration of 32.85 s in the 75 s recording. LFTS (*n* = 182) were the dominant sound produced in bouts (*n* = 15) throughout the event at a rate of 2.4 calls per/s (145.6 caLL per/min). All LFTS were produced when the dolphin was pursuing an individual trevally, until prey remains were regurgitated. There was an average of 12.1 ± 7.4 calls produced per bout and bouts ranged from 2 to 27 calls (Figure 3). LFTS had a mean duration of 88.5 ± 16.3 ms (range = 42.7–140.30; median = 87.7). Inter‐signal interval between LFTS was 87.4 ± 5.5 ms (range = 78.3–97.7). The mean peak frequency of tonal calls was 498.5 ± 98.7 Hz and ranged from 301.5 to 904.4 Hz. Between bouts of LFTS, the dolphin produced echolocation bouts (*n* = 18) of a mean duration of 0.88 ± 0.51 s and range of 0.36–2.14 s.

## 4 Discussion

This study provides observations of a novel foraging tactic in a Common Bottlenose Dolphin. The repeated observations of the same sequence of foraging behaviours suggest that this tactic is regularly employed by this individual with the purpose of inducing regurgitation in the trevally, and several characteristics of these interactions are consistent with facultative kleptoparasitism. As such, we propose that these observations represent the first direct evidence of kleptoparasitism in a cetacean.

A key prerequisite of kleptoparasitism is that there should be a net gain from stealing food that is greater than by individual foraging (Brockmann and Barnard 1979). The profitability of stealing food in this case is unclear without additional data on the dolphin's foraging behaviour. However, the observed interactions have several characteristics that are important in the development of kleptoparasitism: a large concentration of hosts with predictable habits, and an ability by the thief to recognise the opportunity for kleptoparasitism and to exploit that resource (Brockmann and Barnard 1979; Iyengar 2008).

The schooling behaviour of the trevally in the same location provides a large and predictable concentration of hosts that can be exploited for food. The repeated sequence of pursuit by the dolphin, followed by the regurgitation by the trevally, suggests that the fish are expelling ingested food as a reaction to being chased. This raises the question as to whether the dolphin is eating regurgitate as an opportunistic act during prey pursuit, or if it is a specialised tactic that has been developed by this individual, with the intention of eating the regurgitate itself. Bottlenose dolphins in other locations (e.g., Western Australia, Florida, Comoros archipelago) are known to feed on several species of trevally (*Caranx* sp. and *Pseudocaranx* sp.) (Berens McCabe et al. 2010; Kiszka et al. 2014; McCluskey et al. 2021), and it is well documented that predator–prey associations can lead to kleptoparasitism (Brockmann and Barnard 1979). The repeated observations of the dolphin's thieving behaviour over several years support the hypothesis that this is a learned behaviour rather than opportunistic exploitation during hunting, particularly as this individual has never been observed predating the trevally directly.

We hypothesise that kleptoparasitism targeting large predatory fish like Bigeye Trevally may be more profitable than foraging directly on the small pelagic fish species that trevally consume, as predation of these species typically requires collective efforts of multiple individuals in small delphinids (Austin et al. 2019; Flower et al. 2012; Gowans et al. 2007; Yates and Broom 2007). The individual observed in this study has never been seen foraging with other dolphins or using tactics other than kleptoparasitism, suggesting that it could be a behaviour that was innovated by this individual rather than adopted through social learning. However, the individual has been observed interacting with trevally in the presence of other dolphins on one occasion, so the latter cannot be completely excluded. Learning has been suggested to be important for an animal's ability to assess the trade‐offs of stealing food rather than self‐foraging. Kleptoparasitic foraging in this individual was first reported anecdotally in December 2017 and it is possible that the dolphin adapted a learned social parasitism strategy for individual foraging. Social learning has been documented in Common Bottlenose Dolphins and is defined as a cultural behaviour that allows transmission of information, either vertical or horizontal, between individuals (Wild et al. 2020). It is possible that kleptoparasitic behaviour is widespread in delphinids and has been unreported in the literature due to difficulties associated with observing underwater hunting behaviour.

**FIGURE 4:**
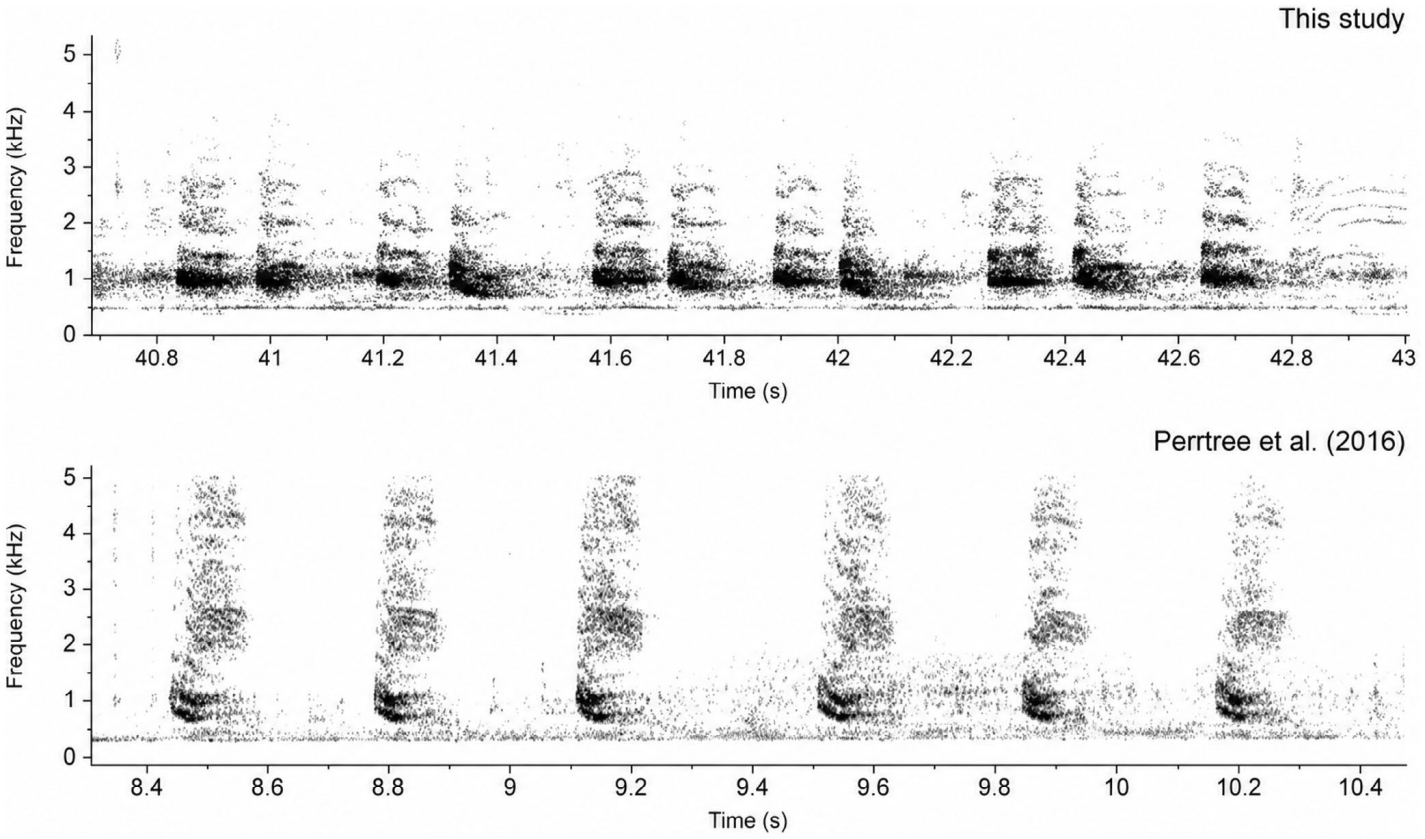
Comparison of spectrograms of low‐frequency tonal sounds recorded in the Common Bottlenose Dolphin ( *Tursiops truncatus* ) in this study and calls reported by Perrtree et al. (2016).

An important prerequisite for kleptoparasitism is that the thief can observe the food and recognise the opportunity to steal it (Iyengar 2008). It is unclear from our observations if the dolphin was able to determine which trevally were suitable targets for inducing regurgitation. However, the scanning behaviour observed at the beginning of each foraging attempt suggests that echolocation could play a role. Delphinids have been shown to use echolocation to differentiate between species of fish, as the varied morphologies of fish swim bladders produce differently structured echoes (Au et al. 2010; Yovel and Au 2010). Dolphins can discriminate small differences in target shape, size, composition and structure (Au and Pawloski 1992). It is therefore possible that the gut content of individual Bigeye Trevally might be detectable, allowing the dolphin to selectively target fish that are more likely to yield the greatest prey intake. It is also possible that sound may play a role in initiating prey regurgitation based on our video and acoustic data. However, additional evidence is needed to support this hypothesis, as our dataset remains limited. The dolphin produced intense echolocation click trains and LFTS that possibly increased stress in the pursued individual trevally. However, it must be acknowledged that sample size was limited and that additional sampling will be needed to more appropriately investigate the function of LFTS, particularly during kleptoparasitic interactions. It is interesting to note that the structure and production of these low‐frequency calls is similar to those reported in previous studies (Perrtree et al. 2016; Simard et al. 2011). During observations of a wild birth and attempted infanticide event in Common Bottlenose Dolphins near Tybee Island in Georgia, USA, similar bouts of low‐frequency calls and a high number of calls during agonistic interactions between multiple animals and the mother‐calf pair were recorded (Figure 4) (Perrtree et al. 2016). Our results, however, suggest that LFTS could be used during foraging but possibly have other functions that need to be further investigated.

This study provides the first direct observations of kleptoparasitism as a foraging tactic adopted by a delphinid. The video and acoustic evidence presented here shows a clear repeated behavioural sequence that leads to an individual of one species (Common Bottlenose Dolphin) stealing food from another (Bigeye Trevally). More directed studies will be needed in the future to investigate whether other dolphins are involved in kleptoparasitism in the waters of the southern Great Barrier Reef, as the prevalence of this tactic is potentially underestimated in cetaceans due to the difficulty of detecting it from surface observations. If other individuals are found to engage in kleptoparasitism, transmission mechanisms of this foraging tactic should be investigated (horizontal vs. vertical transmission). To understand the benefit of this foraging tactic, in‐depth dietary analyses are also warranted. Overall, the foraging behaviour of cetaceans remains underappreciated, and the use of technologies such as animal‐borne video camera tags and unmanned aerial vehicles (drones) are likely to generate new insights into the cryptic behaviours of these elusive marine predators.

## Author Contributions

**Romney Edwards‐Francis:** conceptualization (supporting), data curation (lead), formal analysis (lead), investigation (lead), methodology (lead), resources (supporting), visualization (lead), writing – original draft (lead), writing – review and editing (lead). **Jeremy J. Kiszka:** data curation (supporting), formal analysis (supporting), investigation (supporting), methodology (supporting), visualization (supporting), writing – original draft (supporting), writing – review and editing (supporting). **Michael Ingram:** data curation (supporting), investigation (supporting), methodology (supporting), writing – original draft (supporting), writing – review and editing (supporting). **Pieter Demmers:** data curation (supporting), investigation (supporting), methodology (supporting), writing – original draft (supporting), writing – review and editing (supporting). **Jacinta Shackleton:** data curation (supporting), methodology (supporting), writing – review and editing (supporting). **Eric A. Ramos:** data curation (supporting), formal analysis (supporting), investigation (supporting), methodology (supporting), visualization (supporting). **Asia O. Armstrong:** conceptualization (lead), data curation (supporting), formal analysis (supporting), investigation (supporting), methodology (supporting), project administration (supporting), resources (lead), visualization (supporting), writing – original draft (supporting), writing – review and editing (supporting), supervision (lead).

## Funding

This work was supported by Great Barrier Reef Foundation.

## Conflicts of Interest

The authors declare no conflicts of interest.

## Data Availability

All the required data are uploaded to an online repository as Supporting Information. A link to the video files is here: https://doi.org/10.5281/zenodo.21973777.
